# Editorial: Computer vision and human behaviour to recognize emotions

**DOI:** 10.3389/fpsyg.2024.1441678

**Published:** 2024-06-25

**Authors:** Tindara Caprì, Giovanni Cicceri, Salvatore Distefano, Gennaro Tartarisco

**Affiliations:** ^1^Department of Life and Health Sciences and Health Professions, Link Campus University, Rome, Italy; ^2^Department of Biomedicine, Neuroscience and Advanced Diagnostics (BiND), University of Palermo, Palermo, Italy; ^3^MIFT Department, University of Messina, Messina, Italy; ^4^Institute for Biomedical Research and Innovation (IRIB), National Research Council (CNR), Messina, Italy

**Keywords:** artificial intelligence, machine learning, computer vision, human behavior, emotion mining, general psychology

Computer vision is the interdisciplinary scientific field that deals with how computers can gain high-level understanding from digital images or videos. From the perspective of engineering, it seeks to automate tasks that the human visual system can perform. Additionally, integrating insights from general psychology and computer vision can enhance our understanding of human behavior and the mental processes underlying emotions and future actions (Wiley and Lucas, 2018; Patel and Patel, 2020). The present Research Topic aimed to discovery and argued computer vision solutions suitable for recognizing and managing emotions, considering the latest research contributions in the multidisciplinary field of engineering, neuroscience and behavioral psychology applied to the field of computer vision.

A study, included in this Research Topic, carried out a cross-racial validation of two common facial emotion recognition system, respectively FaceReader and DeepFace (Li et al.). FaceReader was a user-friendly and versatile software for examining face images or videos. This system employes deep-learning models to detect faces and classify facial expressions. DeepFace was a Python package that implements face recognition and facial attribute analysis, such as: age, gender, emotion, and race. It used a different models based on convolutional neural networks. To compare and validate these two systems, the authors used two Western and two Eastern face datasets and calculated the accuracy for each system on face images from the same emotion category of each dataset. With reference to the Western dataset both systems obtained a high accuracy, whereas for the Eastern dataset accuracy was poor.

A second study of Kim et al. also investigated emotion recognition comparing human behavior and machine analysis in three featural parameters, such as prototypicality, ambiguity, and complexity. The authors carried out two studies in which facial expression videos and related images depicting the peak of the target and non-target emotion were presented to both human observers and the machine classifier. Results were interesting because it was found that recognition performance by the machine was better to humans for both target and non-target images.

Another study of Zhang et al. investigated facial emotion recognition in virtual reality environments, using a novel system with MobileNet V2. This system was a lightweight convolutional neural network appropriate for running on virtual reality headsets. Obtained results were controversial because the model better recognized some emotions like “Neutral,” “Happiness,” “Sadness,” and “Surprise,” but the model confused “Anger” and “Fear” emotions with “neutral” ones.

In the last study included in this Research Topic, the authors carried out a quasi-experimental study aimed to identify children's accuracy in recognizing basic and neutral facial emotions in two conditions (Mastorogianni et al.). A condition was no-masks on the face and another condition was faces partially covered by various types of masks. Results showed that children accurately recognized emotions even when face was covered by mask.

The present Research Topic shows that the intersection of computer vision and human behavior opens promising possibilities for emotion recognition, with applications that can profoundly impact various fields. As the technology advances, addressing the challenges and ethical issues will be paramount to ensure that emotion recognition systems are reliable, unbiased, and respectful of individual privacy. With responsible development and deployment, computer vision could revolutionize our understanding and interaction with human emotions, leading to more empathetic and responsive systems.

## Author contributions

TC: Writing – original draft, Writing – review & editing. GC: Writing – original draft, Writing – review & editing. SD: Writing – original draft, Writing – review & editing. GT: Writing – original draft, Writing – review & editing.
